# Effects of Pork Backfat Replacement with Emulsion Gels Formulated with a Mixture of Olive, Chia and Algae Oils on the Quality Attributes of Pork Patties

**DOI:** 10.3390/foods12030519

**Published:** 2023-01-23

**Authors:** Nicoleta Cîrstea (Lazăr), Violeta Nour, Andrei Iulian Boruzi

**Affiliations:** 1 Faculty of Food Science and Engineering, Dunărea de Jos University of Galati, Domnească Street 111, 800201 Galati, Romania; 2 Department of Horticulture & Food Science, University of Craiova, 13 AI Cuza Street, 200585 Craiova, Romania

**Keywords:** reformulated pork patties, emulsion gels, gelatin, chitosan, fatty acid profile, technological properties

## Abstract

This paper reports on the development of new emulsion gels containing a mixture of olive, chia and algae oil emulsified with soy protein isolate and stabilized by two different cold gelling agents, gelatin (EGEL) and chitosan (ECHIT), and to evaluate their potential use as pork backfat replacers in cooked pork patties. Reformulated patties were produced by half and full pork backfat replacement and compared to normal fat patties and reduced fat content patties made by replacing half of the added fat with water. Color parameters, pH and thermal stability of the emulsion gels were determined at processing and after 10 days of refrigerated storage. Proximate composition, fatty acid profile, technological properties and sensory attributes were evaluated after patty processing, while color parameters, pH and lipid oxidation were monitored in patties during 15 days of refrigerated storage (4 °C). Reformulated patties showed significant improvements of the lipid profile (lower saturated fatty acid content and *n*-6/*n*-3 ratio and higher long-chain polyunsaturated fatty acid content) as compared to the controls. In terms of technological properties, chitosan was more effective than gelatin as a stabilizer of the emulsion gel. All reformulated patties showed a good evolution of lipid oxidation during storage and acceptable sensory attributes.

## 1. Introduction

The recent advances in nutrition science and the societal efforts to implement nutritional education have led consumers to be increasingly aware of the diet–health relationship and more focused on opting for healthier diets and lifestyle [1]. The epidemiological studies have shown that higher SFA intake leads to the increase in the low-density lipoprotein (LDL) cholesterol, which, in turn, represents a risk factor for developing cardiovascular disease and type II diabetes [2]. As a result, the dietary guidelines have promoted the decrease in saturated fatty acids (SFA) intake and the replacement of SFA with polyunsaturated fatty acids (PUFA) [3,4]. In addition, there is a great body of evidence from biochemical, epidemiological and clinical studies that longer-chain *n*-3 polyunsaturated fatty acids (*n*-3 PUFAs), mainly eicosapentaenoic (EPA) and docosahexaenoic (DHA) acids, exert strong beneficial health effects by modulation of eicosanoid synthesis, thus decreasing the risk of cardiovascular diseases and certain cancers [5]. Numerous studies demonstrated that overconsumption of *n*-6 polyunsaturated fatty acids (*n*-6 PUFAs) associated with lower intake of *n*-3 PUFAs contributes to the development of many modern chronic inflammatory diseases, cardiovascular diseases, cancers and autoimmune diseases [6]. The importance of reducing the *n*-6/*n*-3 ratio in the human diet is mainly related to the anti-inflammatory functions of *n*-3 PUFAs, and especially of linolenic acid and longer-chain *n*-3 PUFAs [2]. To meet consumer demands for healthier food products, the food industry is challenged to develop new products with reduced energy density, saturated fat and cholesterol and with improved fatty acid profile [7]. In the meat industry, one of the main approaches is to reformulate the meat products by replacing the animal fat with oils of plant and marine origin that better meet the current nutritional recommendations [8]. However, the animal fat from fat-rich meat products acts as a structuring and tasting agent, thus imparting specific texture, taste and appearance in the traditional meat products which are popular among consumers [9]. As a result, the animal fat replacement could negatively impact on the technological and sensory features of the meat products, especially on their texture and flavor, thus reducing the consumer acceptability [7,10]. In addition, the higher unsaturation degree of the lipids in the vegetable and marine oils increases their susceptibility to undergo oxidation, thus limiting their use in meat products [11]. In order to avoid these problems, the research from the meat industry created and investigated a variety of gel structures, such as oleogels and emulsion gels, able to immobilize the oil droplets and to mime the firmness and water-holding capacity of pork backfat used in most of the meat products [12,13]. In addition, the results revealed the capacity of these solid oil structured systems to protect the incorporated lipids from oxidation [14].

An emulsion gel is characterized by a gel-like network structure made from a protein-stabilized emulsion by incorporation of a hydrocolloid stabilizer during emulsification or other ingredients, such as proteins or polysaccharides, after emulsion formation. Its structure was described as a continuous network which combines aggregated emulsion droplets and cross-linked biopolymer molecules [8]. The emulsion gels can be prepared by heat setting, acid- or enzyme-induced gelation [15] or by adding cold gelling agents such as polysaccharides, proteins or their combination [16,17,18,19,20].

Chitosan is a versatile low-cost biopolymer with a great gel-forming ability due to its susceptibility to form intra- and inter-molecular hydrogen bonds and to undergo chemical modifications ascribed to the presence of diverse functional groups [21]. Although some flat chitosan structures (films and coatings) have been widely applied in food packaging, to our knowledge, it was never used as a stabilizer in the emulsion gels incorporated as fat replacers in meat products. As for the use of proteins, some previous studies successfully replaced pork backfat in meat products by oil-in-water emulsions stabilized by various protein systems formulated using sodium caseinate, soy protein isolate, gelatin, meat protein and microbial transglutaminase [5,13,22]. Olive and chia oils have been previously incorporated individually or together in oleogels or emulsion gels used as pork fat replacers to obtain healthy meat products [8,23]. The high content of monounsaturated fatty acids (mostly oleic acid) and naturally occurring antioxidants makes olive oil a good candidate to be used as animal fat replacer in meat products. Chia oil also shows a beneficial fatty acid profile due to its high content of polyunsaturated fatty acids (>80%), of which around 60% is α-linolenic acid (18:3*n*-3) [24]. As a result, it could be successfully incorporated in oleogels or emulsion gels in order to increase the *n*-3 PUFAs content of meat products.

Algae oil is an excellent source of long-chain *n*-3 PUFAs, namely EPA and DHA, which are associated with an array of health benefits but whose dietary availability is limited [25]. Hence, using algae oil as an ingredient in animal fat replacers could have a major positive impact on the nutritional profile of the reformulated meat products by enhancing their *n*-3 fatty acid composition.

The present study aimed to develop gelled emulsions formulated with a mixture of olive, chia and algae oils emulsified with soy protein isolate and stabilized by two different cold gelling agents, gelatin and chitosan, and to evaluate their potential use as pork backfat replacers in cooked pork patties. Nutritional, technological and sensory properties of reformulated patties were evaluated, as well as their oxidation stability during 15 days of refrigerated storage. Normal fat and reduced fat content patties were used as controls.

## 2. Materials and Methods

### 2.1. Materials and Reagents

Patties were prepared with fresh postrigor meat (mixture of *biceps femoris*, *semimembranosus*, *semitendinosus*, *gracilis* and adductor muscles, 12 kg) and pork backfat (4 kg) purchased from a local meat market. Algae oil was provided by Fair Trade Handels AG (Luzern, Schweiz), a commercially available oil obtained from *Schizochytrium* sp. (Herkunft, USA), with natural blood orange aroma and rosemary extract as antioxidant, with the following nutritional specification for lipid composition: omega-3 fatty acids 1116 mg/2.5 mL, thereof DHA 700 mg/2.5 mL, EPA 350 mg/2.5 mL and DPA 35 mg/2.5 mL. The extra virgin olive oil was from Monini brand (Spoleto, Italy), the lipid fraction containing 15.2% SFA, 75% MUFA and 9.8% PUFA, according to the information provided by the supplier. The extra virgin chia oil was from Huilerie de Lapalisse (Lapalisse, France) and had the following nutritional specification for lipid composition: SFA, 10.7%; MUFA, 9.5%; and PUFA, 79.8%. Soy protein isolate was provided by Solae Belgium N.V. (Ieper, Belgium), gelatin by Top Ingrediente S.R.L. (Sat Bacu, Romania) and chitosan was obtained from BiOrigins (Fordingbridge, UK). Thiobarbituric acid, trichloracetic acid and malondialdehyde were obtained from Sigma-Aldrich (St. Louis, MO, USA). All other chemicals used were of analytical grade and were purchased from Merck (Darmstadt, Germany).

### 2.2. Preparation of Emulsion Gels

Two different types of O/W emulsion gels were formulated and used in patties as pork backfat replacers, both prepared with stabilizer systems based on soy protein isolate and a cold gelling agent: (1) gelatin (EGEL) and (2) chitosan (ECHIT). Both emulsion gels had the same oil content (45%) consisting of a mixture of 45% olive oil, 35% chia oil and 20% algae oil. 

Emulsion gels were prepared one day before the processing of patties according to the method described by Pintado and Cofrades [13]. Briefly, preparation of emulsion gels consisted of homogenizing the soy protein isolate (10%) with water (42%) at high speed in a BOSCH VitaBoost MMBH6P6 blender (Bosch, Stuttgart, Germany) (30 s, approx. 5600 rpm), then adding the cold gelling agent (3%) and continuing to mix for 15 s. Finally, the mixture was homogenized in the same blender with gradual addition of the oil mixture (45%) described previously. The emulsion gels were placed in plastic containers, stored under refrigeration (4 °C) and used within 24 h. No syneresis was noticed during storage and meat patty processing. Each emulsion gel was prepared in duplicate. Color, pH and thermal stability of emulsion gels were assessed immediately after being obtained, as well as after 10 days of refrigerated storage (4 °C).

### 2.3. Thermal Stability and pH of Emulsion Gels

Thermal stability, in terms of water and oil binding properties, of the emulsion gels was evaluated in triplicate according to the method of Jiménez-Colmenero et al. [22] with minor modifications. About 25 g emulsion samples were introduced into tubes, which were hermetically sealed and heated at 70 °C in a water bath for 30 min. After heating, the tubes were centrifuged at 2500× *g* (Hermle Z300, Hermle Labortechnik, Wehingen, Germany) for 15 min; then, they were opened and left standing upside down for 50 min to drain out the separated fat and water (exudate) onto a plate. The remaining emulsion was cooled, weighed and the emulsion stability, as total fluid release (TFR), was expressed as percent of initial sample weight.

The pH was determined in triplicate at room temperature on a Hanna pH meter HI255 (Hanna Instruments, Padova, Italy) in emulsion homogenates prepared by mixing the emulsion in water in a ratio of 1:10 (*w/v*).

### 2.4. Preparation of Pork Patties

Six different patty formulations were developed as described in Table 1. Two formulations without replacement of pork backfat were prepared as references, one with normal (PBF1/1) and the other with half pork backfat content (PBF1/2). In addition, four reformulated patties were made by partial and total replacement of pork backfat with emulsion gel: two in which pork backfat was half replaced by EGEL (EGEL1/2) or ECHIT (ECHIT1/2) and another two formulated by total replacement of pork backfat with EGEL (EGEL1/1) and ECHIT (ECHIT1/1).

The meat was trimmed of separable fat and connective tissues. Both the pork meat and the pork backfat were cut into small pieces and minced in a meat grinder BOSCH ProPower MFW68660 (Bosch, Stuttgart, Germany) through a 3 mm plate. The salt, water and emulsion gel were added and the mixture was hand kneaded for 10 min. Then, the mixtures were divided into raw patties (50 g), which were shaped by compressing in a Petri dish (565 mm diameter and 180 mm depth). Thirty patties were made for each experimental variant. The samples were placed in an electric oven (Beko, BIM24300GPS, Turkey) preheated for 15 min at 180 °C and allowed to cook for 25 min until the meat reached 75 ± 1 °C in the center. After cooling to room temperature, the patties were packed in polyethylene bags under aerobic conditions and stored at 4 ± 1 °C for 15 days. The whole experiment was performed in triplicate.

The proximate analysis, fatty acid composition and sensory evaluation was performed on cooked patties immediately after processing (day 0). Determination of pH, color and thiobarbituric acid reactive substances (TBARS) as a measure of lipid oxidation was carried out at the beginning (day 0) and after 5, 10 and 15 days of storage.

### 2.5. Color Measurement

The color of the patties was measured just after manufacturing and after 5, 10 and 15 days of storage on a homogenized ground mixture of each sample. A PCECSM1 colorimeter (PCE Instruments, Southampton, UK) operating in the CIEL*a*b* system with spectral reflectance was used, calibrated against a white standard provided with the instrument. The color co-ordinates L* (brightness), a* (+redness/–greenness) and b* (+yellowness/–blueness) were measured on three samples from each variant at five points on each sample.

### 2.6. pH Measurement

The pH of pork patties was measured on the sample homogenates using a digital pH meter (Hanna HI255, Padova, Italy). The homogenates were obtained by mixing 5 g of sample in 50 mL of distilled water by using a vertical blender (Braun MQ5137BK, 750W). Two homogenates were prepared for each sample, of which two analyses were performed.

### 2.7. Proximate Analysis

The patties were analyzed in triplicate for moisture, fat and protein content according to AOAC procedures [26]. Moisture content was determined based on moisture loss at 105 °C in a drying oven (Memmert ULM500, Uden, The Netherlands), the fat content was determined by the Soxhlet method using a Soxhlet automatic extraction system (SER 148/3, Velp Scientific, Usmate, Italy), the protein content by the Kjeldahl method using an automated nitrogen analyzer (UDK 149 Velp Scientific, Milan, Italy) and the ash content using a Caloris CL 1206 oven (Romania). The energy value was calculated based on 9.1 kcal/g for fat and 4.1 kcal/g for carbohydrates and protein. The total carbohydrate content was determined by difference according to the equation:

Carbohydrates (%) = 100 − (% protein + % fat + % ash + % moisture)(1)

### 2.8. Technological Properties

The technological properties were evaluated in triplicate using three samples for each formulation. The raw patties as well as the cooked patties were weighed and their diameter was measured by vernier caliper at three different places. The cooked patties were evaluated after cooling to room temperature (25 °C). Cooking loss was calculated by the weight difference between raw and cooked patties and expressed as a percentage of the initial weight (Equation (2)). The shrinkage due to cooking was determined by the diameter difference between raw and cooked patties and expressed as a percentage of the initial diameter (Equation (3)) [27]. The moisture retention and the fat retention, representing the amount of moisture or fat retained in the cooked product per 100 g of raw sample, were determined using Equations (4) and (5), respectively [28].

Cooking loss (%) = [(weight_raw_ − weight_cooked_)/weight_raw_] × 100 (2)

Shrinkage (%) = [(diameter_raw_ − diameter_cooked_)/diameter_raw_] × 100 (3)

Moisture retention (%) = [(100 − cooking loss (%)) × moisture_cooked_]/100 (4)

Fat retention (%) = [(weight_cooked_ × fat_cooked_)/(weight_raw_ × fat_raw_)] × 100 (5)

### 2.9. Fatty Acid Analysis and Nutritional Indices

Fatty acid content from cooked patties was determined in triplicate in the lipid extracts by fatty acid methyl ester (FAME) gas chromatography using a gas chromatograph Perkin-Elmer Clarus 500 (Shelton, MA, USA). The fatty acids from patty samples were converted to their methyl esters (FAME) by transesterification at 80 °C in methanol containing 3% concentrated sulfuric acid. Methyl esters of fatty acids were separated on a DB-23 GC capillary column (60 m × 0.25 mm id × 0.25 µm film thickness) from Agilent J&W GC Columns (Santa Clara, CA, USA) and detected with a flame ionization detector (FID). The column temperature increased at 5 °C/min from 180 °C to 220 °C. Hydrogen was the carrier gas with a linear velocity of 35 cm/s at 180 °C. FAME identification was carried out by comparing retention times with those of the individual standards from Sigma-Aldrich Chemical Co. (MO, USA). The results were expressed as grams of fatty acid per 100 g total fatty acids. The average amount of each fatty acid was used to calculate the sum of the total saturated (Σ SFA), total monounsaturated (Σ MUFA), total polyunsaturated (Σ PUFA), total *n*-3 polyunsaturated (Σ *n*-3 PUFAs), total *n*-6 polyunsaturated (Σ *n*-6 PUFAs) fatty acids, and sum of eicosapentaenoic and docosahexaenoic acids (EPA + DHA).

The *n*-6/*n*-3 ratios was considered in order to evaluate the quality of the fatty acids profile. In addition, the atherogenicity (AI) and thrombogenicity (TI) indexes were calculated as presented by Equations (6) and (7), respectively, as stated by Ulbricht and Southgate [29], and the ratio of hypocholesterolemic and hypercholesterolemic fatty acids (h/H) was calculated as defined by Equation (8) according to Santos-Silva et al. [30].

AI (Atherogenic Index) = (C12:0 + 4 × C14:0 + C16:0)/(Σ MUFA + Σ PUFA)(6)

TI (Thrombogenic Index) = (C14:0 + C16:0 + C18:0)/(0.5 × Σ MUFA + 0.5 × Σ PUFA *n*-6 + 3 × Σ PUFA *n*-3 + PUFA *n*-3/PUFA *n*-6) (7)

h/H (hypocholesterolemic/Hypercholesterolemic index) = (C18:1 *n*-9 + C18:2 *n*-6 + C20:4 *n*-6 + C18:3 *n*-3 + C20:5 *n*-3 + C22:5 *n*-3 +C22:*n*-3)/(C14:0 + C16:0) (8)

### 2.10. Lipid Oxidation

The patties were assessed for oxidative stability by determining the evolution of the thiobarbituric acid reactive substances (TBARS) value during 15 days refrigerated storage. TBARS values of pork patties were determined in triplicate for each formulation at day 0 and after 5, 10 and 15 days of refrigerated storage according the method described by Witte et al. [31] with minor changes. Briefly, 5 g of each sample was extracted in 12.5 mL of 20% trichloroacetic acid with intense stirring in a vortex, then transferred to a 25 mL volumetric flask and diluted up to the volume with cold distilled water. The solution was filtered (Whatman No. 1, Merck, Darmstadt, Germany) and 5 mL of the supernatant was mixed with 5 mL of 0.02 M 2-thiobarbituric acid and heated at 100 °C for 35 min. After cooling in an ice bath to room temperature, the absorbance was read at 532 nm with a Varian Cary 50 UV spectrophotometer (Varian Co., Palo Alto, CA, USA). TBARS values were expressed as mg malonaldehyde (MDA) per kg of patty sample.

### 2.11. Sensory Analysis

The sensory attributes of pork patties (color, taste, flavor, texture and overall acceptability) were evaluated after patty processing on a 9-point hedonic scale [32], ranging from 1 = “dislike extremely” to 9 = “like extremely”. Twelve panelists selected from the staff of the Department of Food Science from University of Craiova participated in the sensory analysis session. The cooked patties were coded, heated for 15 s in a microwave, and then presented to the panel for evaluation. The panelists received six randomized samples per session with a 10 min interval between samples to reduce fatigue. They were invited to clean their mouth palate with bread and water between samples and to make any relevant comments about their sensory perception of the samples.

### 2.12. Statistical Analysis

The statistical analysis of data was conducted using the Statgraphics Centurion XVI software (StatPoint Technologies, Warrenton, VA, USA). One-way analysis of variance (ANOVA) was performed to evaluate the statistical significance (*p* < 0.05) of the effect of patty formulation in proximate and fatty acid composition, technological properties and sensory attributes. Additionally, two-way ANOVA as a function of formulation and storage time was performed for pH values, color parameters and TBARS values. Differences between means were evaluated with the least significant difference (LSD) test at 5% level of probability.

## 3. Results and Discussion

### 3.1. Color, pH and Thermal Stability of the Emulsion Gels

Color of the emulsion gel is an important parameter, as well as its evolution during storage, because the color modifications could entail color differences in the food matrix where the emulsion gel is added. Just after obtaining the emulsion gels, significantly higher (*p* < 0.05) L* values were found for ECHIT than for EGEL (Table 2). L* values decreased during storage in both emulsion gels and, after 10 days of refrigerated (4 °C) storage, similar L* values were found for ECHIT and EGEL. Redness (a* values) was also significantly reduced (*p* < 0.05) during storage in both emulsion gels but remained significantly higher (*p* < 0.05) in ECHIT as compared with EGEL, both initially and at the end of 10 days of refrigerated storage.

The b* values remained in similar values (*p* > 0.05) during storage for EGEL, while they decreased for ECHIT, so that, after 10 days of refrigerated storage, b* values were lower for ECHIT than for EGEL. Similar b* values were reported for a gelled emulsion containing carrageenan and algae oil developed as animal fat replacer by Alejandre et al. [32]. They attributed the high b* values of the gels to the carotenoids present in the algae oil. The decrease in the yellowness (b* values) during storage was previously related to the isomerization and to the oxidative degradation of carotenoids [33], and these processes could also be the reason of the decrease in redness (a* values) in the present study. The modifications of color parameters during storage were higher in ECHIT as compared to EGEL, demonstrating a greater color stability of EGEL.

Figure S1 in the supplementary material presents images of the appearance of gelatin (EGEL) and chitosan (ECHIT) emulsion gels immediately after processing.

The pH value is an important parameter affecting the physico-chemical, biochemical, technological and microbiological features of the meat product. The pH value was significantly higher (*p* < 0.05) in ECHIT than in EGEL throughout storage (Table 2). A higher pH was previously associated with a higher water-holding capacity in meat products [34,35]. The pH of emulsion gels increased slightly during storage in both emulsion gels as a result of the alkaline nitrogenous compounds formed through increased protein degradation. No syneresis or exudate release was noticed during processing or 10 days refrigerated storage in both emulsion gels. Previous studies reported also good phase separation stability of gelled emulsions stabilized by proteins (caseinate and gelatin soy protein) cross-linked or not with transglutaminase [8,36] or polysaccharides [12].

The thermal stability of emulsion gels is a technological feature of interest considering their use in cooked meat products. Both emulsion gels presented excellent thermal stability both initially and after 10 days of storage; however, ECHIT showed significantly (*p* < 0.05) higher thermal stability than EGEL. Good thermal stabilities have been also previously reported for oil-in-water emulsion gels stabilized by soy protein isolate used in the reformulation of meat products [22,37].

### 3.2. Proximate Composition and Energy Values

The pork backfat reduction or its replacement with emulsion gels affected the proximate composition and energy value of cooked patties. As expected, the replacement of half of the fat content with water in PBF1/2 led to a significant increase in the moisture content of cooked patties (by 15.7%) as compared to the control ones (PBF1/1) (Table 3). Likewise, the replacement of pork backfat with emulsion gels led to increases in the moisture content (in the range of 9.1 to 16.4%). The ECHIT1/1 samples showed the highest moisture content among all variants, with a percentage of 61.69%, while the controls (PBF1/1) exhibited the lowest moisture content (53.00%). As previously presented, the emulsion gels were made with 42% water; hence, the higher moisture content of the ECHIT and EGEL reformulated patties may be due to the high moisture content of the gels used to substitute the pork backfat.

Several previous studies reported also the increase in the moisture content in reformulated patties after incorporation of gel systems with the aim to replace the animal fat with vegetable oils [13,14,32] and noticed the role of these emulsions as a source of water to reformulated meat products [38,39]. In the present study, the total replacement of pork backfat with emulsion gels (EGEL1/1 and ECHIT1/1) led to moisture content values that did not significantly (*p* > 0.05) differ from that of the formulation with half pork backfat content (PBF1/2).

The highest fat content was found in PBF1/1 (control) patties (21.47%), while replacing half of the fat content with water (PBF1/2) determined a considerable level of fat reduction (36.1%) (Table 3). The reformulated patties with half replacement of pork backfat showed a considerable fat reduction in their composition (21.23% and 23.01% for EGEL1/2 and ECHIT1/2, respectively), while the full fat replacement with emulsion gels resulted in a fat reduction of 39.88% and 36.8% for EGEL1/1 and ECHIT1/1, respectively.

The energy value of the reformulated patties with full replacement of the pork backfat was around 213 kcal/100 g, meaning around 26% of energy reduction in these products. In control patties, around 67.2% of energy value was supplied by fat, while, in reformulated patties, this percentage decreased, reaching 55% and 57% for EGEL1/1 and ECHIT1/1, respectively. Moreover, the energy values of these products do not differ significantly from the energy value of the patties with half pork backfat content (PBF1/2). 

In agreement with the results obtained in the present study, other previous studies reported a significant decrease in the fat content in meat products reformulated by substitution of animal fat with emulsion gels containing various oils [27,32,40,41].

The ash content of the patties (ranging between 2.21 and 2.43%) was only slightly affected by lipid reformulation. Higher ash contents were found in patties incorporating emulsion gels stabilized by chitosan.

No significant difference was found in the protein content of the patties, except the samples reformulated by total replacement of the pork backfat with emulsion gel stabilized by gelatin, which showed significantly (*p* < 0.05) higher protein content. However, other studies reported the decrease in the protein content with the increase in the level of incorporation of emulsion gels in meat products [5,41]. The proteinaceous nature of gelatin versus the polysaccharide nature of chitosan may be the reason for the higher protein content in EGEL 1/1 and EGEL1/2 than in ECHIT1/1 and ECHIT1/2 patties. Some previous studies reported also an increase in protein content of meat systems produced with double emulsions as animal fat replacers using sodium caseinate [42] or whey protein isolate [39] for the emulsion stabilization. Except protein content, no clear effect was observed on proximate composition concerning the type of emulsion gel systems used.

### 3.3. Fatty Acids Profile

The analysis of the fatty acid profile as well as the evaluation of the nutritional indices of the reformulated products are essential to prove their nutritional value and functionality. The fatty acid content of the different pork patty formulations is shown in Table 4. As expected, the addition of emulsion gels made with olive, chia and algae oils as a substitute for pork backfat influenced to a great extent the fatty acid profile. Oleic acid (C18:1*n*-9) was the major fatty acid in all samples; however, it was followed by palmitic (C16:0) and stearic (C18:0) acids in control samples (PBF1/1) and by linolenic (C18:3*n*-3), linoleic (C18:2*n*-6), docosadienoic (C22:2*n*-6) and docosahexaenoic (C22:6*n*-3) acids in the patties reformulated by full substitution of pork backfat with emulsion gels (EGEL1/1 and ECHIT1/1).

The SFA content was reduced by around 44% in patties reformulated by half replacement of pork backfat content with emulsion gels (EGEL1/2 and ECHI1/2) and by about 64.4% in the samples with full replacement (EGEL1/1 and ECHIT1/1) as compared with the control patties (PBF1/1). 

A lower level of MUFA content was also noticed in reformulated patties (by about 15.1% and 32% in samples with half and full replacement, respectively) as compared with the control samples. However, oleic acid remained the main fatty acid in the reformulated patties as in the control patties (Table 4), as a result of its high content both in pork fat and in olive oil that contributed 45% in the lipid phase of the emulsion gels [13,43]. Contrariwise, the PUFA content increased 3.2 times in patties reformulated by partial replacement of pork backfat with emulsion gels (EGEL1/2 and ECHIT1/2) and 4.6 times in those reformulated by full replacement (EGEL1/1 and ECHIT1/1). Similarly, previous studies also reported the reduction in SFA and increase in *n*-6 PUFAs content as a result of the animal fat replacement with emulsion gels based on vegetal oils [7,27,41]. Other studies using fish or algae oils as animal fat substitutes reported the increase in total PUFA and especially long-chain *n*-3 fatty acids in reformulated meat products [32,44].

The dietary recommendations promote reducing saturated fatty acids in the diet as they are associated with the increase in low-density lipoprotein (LDL) cholesterol and with the negative effect on tissue sensitivity to insulin and inflammation. At the same time, they promote increasing the intake of PUFAs in order to decrease the risk of cardiovascular diseases and type II diabetes. However, the quality of PUFAs plays a major role in health effects. The epidemiological studies demonstrated that *n*-3 PUFA and *n*-6 PUFA groups have different effects on the cell growth, proliferation and progression of neoplastic lesions [2]. Generally, the eicosanoids derived from *n*-6 PUFA are proinflammatory, whereas those derived from *n*-3 PUFA are anti-inflammatory. As a result, the high intake of *n*-6 PUFAs creates proinflammatory effects, which may increase the risk of developing several diet-related chronic diseases [45]. In the present Western diet, the level of *n*-6 PUFA is high, while that of *n*-3 PUFA is very low, which resulted in a rise in the *n*-6:*n*-3 ratio to more than 10:1 [46]. 

No significant differences were found between the control samples (PBF1/1) and those with half pork backfat content (PBF1/2) concerning SPA, MUFA, PUFA and PUFA*n*-3 content. However, replacing half of the fat content with water determined a significant (*p* < 0.05) decrease in the PUFA*n*-6 content as well as of the *n*-6/*n*-3 ratio, mainly due to lower values of linoleic and arachidonic acids. PUFA *n*-3 content multiplied also in reformulated patties by 6.78 and 7.41 times in EGEL1/2 and ECHIT1/2 samples, respectively, and by about 14.75 times in EGEL1/1 and ECHIT1/1 patties. PUFA *n*-6 content was also increased by patty reformulation but to a lesser extent compared to PUFA *n*-3; as a result, the *n*-6/*n*-3 ratio significantly (*p* < 0.05) decreased. Control patties (PBF1/1) showed values of the *n*-6/*n*-3 ratio close to 4.5, while this ratio decreased by 2.78–2.90 times in patties reformulated by substitution of half of the pork backfat with emulsion gels (1.62 for EGEL1/2 and 1.55 for ECHIT/2) and by 6.17–6.26 times in those with full substitution (0.73 for EGEL1/1 and 0.72 for ECHIT1/1) (Table 4). The *n*-6/*n*-3 ratio was less than the values considered healthy (1:1 to 2:1) in all reformulated patties, in agreement with the nutritional recommendations [47]. 

No significant differences were found between EGEL1/2 and ECHIT1/2 and between EGEL1/1 and ECHIT1/1, respectively, regarding SPA, MUFA, PUFA, PUFA*n*-3, PUFA*n*-6 content and all calculated nutritional indices.

A positive outcome is the increase in the docosahexaenoic (C22:6*n*-3, DHA) and eicosapentaenoic (C22:5*n*-3, EPA) fatty acids content in the reformulated patties, which is directly related to the fatty acid composition of the algae oil incorporated in the emulsion gels. These long-chain *n*-3 PUFAs have been associated with an array of health benefits against cardiovascular diseases, including hypotriglyceridemic and anti-inflammatory effects, as well as antihypertensive, anticancer, antioxidant, antidepression, antiaging, and antiarthritic effects [48]. 

Chia oil also contributed to the increase in *n*-3 PUFA content of the patties as it consists of up to 68% of *n*-3 PUFAs and 19% of *n*-6 PUFAs, of which linolenic acid is represented in the highest amount [49]. Olive oil has been used previously for the partial substitution of pork backfat in meat products [22] as well as chia oil [13,27] in order to increase the PUFA/SFA ratio and to reduce the *n*-6/*n*-3 ratio, and the reported results were in good agreement with those obtained in the present study.

Clupanodonic acid C22:5*n*-3 (docosapentaenoic acid, DPA) is also a very long-chain *n*-3 PUFA acid and an algal metabolite that was not detected in controls but was quantified in the reformulated patties. DPA is recognized as an essential bioactive fatty acid in view of its intermediary position between eicosapentaenoic acid and docosahexaenoic acid in the *n*-3 synthesis pathway and of its potential health benefits, including anti-inflammatory actions, antiplatelet aggregation and improved plasma lipid profile [50]. 

Both partial and total replacement of pork backfat with emulsion gels significantly improved the healthiness of pork patties by decreasing the atherogenic index (0.62 in control PBF1/1 vs. 0.27 and 0.15 in EGEL1/2 and EGEL1/1, respectively) and thrombogenic index (1.21 in control PBF1/1 vs. 0.32 and 0.12 in EGEL1/2 and EGEL1/1, respectively) and by increasing the h/H ratio, in agreement with the results previously reported in studies on reformulation of meat products by replacing animal fat with vegetable or marine oils [7,41]. The reduction in the AI and TI as well as the increase in the h/H ratio in reformulated patties as compared with all-pork-fat patties is mainly due to the decrease in the SFA (palmitic, myristic and stearic acids), which are well known to have an indirect atherogenic effect due to increasing LDL-cholesterol concentration but, also, a direct effect by activating the inflammation process [51].

### 3.4. Technological Properties

The cooking loss was significantly (*p* < 0.05) higher in reformulated patties based on emulsion gels made with gelatin as a stabilizing agent as compared with the controls (Table 5). However, cooking loss was lowest in patties with 100% replacement of pork backfat by emulsion gels prepared with chitosan (ECHIT1/1), while no significant differences were found in cooking loss between the patties made with half replacement of pork backfat by emulsion gels stabilized by chitosan (ECHIT1/2) and the controls (PBF1/1).

The lower cooking loss values in these samples might be due to the water-holding capacity and water retention properties of chitosan together with those of the soy protein isolate. Carvalho Barros et al. [41] also reported improved cooking yield as a result of the full replacement of fat with tiger nut oil emulsion in beef burgers, while Heck et al. [27] reported a similar behavior in burgers reformulated by replacing 50% of the fat component by microparticles obtained by external ionic gelation containing chia and linseed oils. The highest cooking loss was registered in samples with half pork backfat content (PBF1/2).

The shrinkage was lower only in patties made by replacement of half of the fat content with water (PBF1/2); no significant differences were found among all the other formulations. Ferreira et al. [7] reported also no statistical differences in shrinkage between control goat meat burgers and reformulated burgers made by replacing the pork fat with the same amount of hydrogel emulsion incorporating olive oil or sunflower oil.

Moisture retention after thermal processing is an important technological parameter that affects the juiciness of the product. The highest moisture retention was found in reformulated patties made by full replacement of pork backfat with emulsion gels stabilized by chitosan (ECHIT1/1), followed by those stabilized by gelatin (EGEL1/1), while the lowest moisture retention was found in control patties (PBF1/1). Other previous studies found also higher losses in reduced-fat meat products than in those with normal fat [13]. Jiménez-Colmenero et al. [22] reported no important quantitative variations in fat and water-binding properties in frankfurters reformulated by replacement of pork backfat with different oil-in-water emulsions stabilized by various proteins (sodium caseinate, soy protein isolate, meat protein and microbial transglutaminase) as a result of their excellent thermal stability and good binding properties.

The highest fat retention was found in control patties, while the lowest in reformulated patties made by full replacement of pork backfat with emulsion gels stabilized by gelatin (EGEL1/1). Among reformulated patties, the highest fat retention was found in patties with 100% pork backfat replaced by emulsion gels stabilized by chitosan (ECHIT1/1). Therefore, the use of chitosan as a stabilizer of the emulsion gel contributed to the increase in the moisture and fat retention during thermal processing as compared with gelatin, in good agreement with the higher thermal stability found for ECHIT emulsion gel as compared to EGEL.

### 3.5. Color Properties and pH

Color is an important feature that influences the consumers’ purchasing choices and acceptability. Therefore, reformulation of the patties should not determine considerable modifications of the appearance of the new products [52]. Color parameters of patties were measured immediately after processing as well as during refrigerated (4 °C) storage.

Reformulation of pork patties by replacing pork backfat with emulsion gels determined the darkening (decrease in the L* values) as well as the increase in the b* values as compared to controls (PBF1/1) but it did not entail significant variations (*p* < 0.05) in redness (a* values) (Table 6). Other previous studies reported also the increment of yellowness (b* values) and no effect on redness (a* values) as a result of the replacement of animal fat with emulsion gels based on vegetable oils [13,41,53]. The increment of b* values may be attributed to the yellowish shade of the incorporated oils, as reported in other studies [7,8,22], as well as of the soy protein isolate used in emulsion gel preparation. 

Replacement of half of the fat content with water (PBF1/2) increased lightness (L* values) and significantly decreased a* values in patties as compared with control patties. ECHIT1/1 samples recorded the lowest L* and the highest b* values among all formulations, in good agreement with the lower L* values and higher b* values found for ECHIT as compared with EGEL. During storage, the b* values increased, while a* values decreased in all formulations. After 15 days of storage, the total diminution of a* values was lower in samples reformulated with emulsion gels stabilized by chitosan as compared with those stabilized by gelatin. The evolution of a* values during storage is relevant since the redness parameter is accounted as the most indicative of meat color acceptability [54]. Therefore, the replacement of pork backfat with emulsion gels incorporating olive, chia and algae oils led to patties that maintain redness and present less changes in color parameters after 15 days of refrigerated storage as compared to the reduced fat patties.

Images of the appearance of control and reformulated patties immediately after processing are shown in Figure S2, available in the “Supplementary Materials” section.

Just after processing, the pH values in the control patties were lower than in any of the reformulated products. However, no significant differences were found among formulations regarding their pH, except the pH of ECHIT1/1, which was significantly (*p* < 0.05) higher (Table 6). Such small pH differences (lower than 0.08 units) between meat products reformulated by replacing animal fat with emulsion gels and controls were also reported in previous studies [7,22]. It was noticed that the pH of the patties reformulated by partial or total substitution of pork backfat with emulsion gels stabilized by chitosan was higher than the corresponding ones made with gelatin. This was expected, as the pH of ECHIT emulsion gels was significantly higher than that of EGEL. The pH slightly increased during 15 days of refrigerated storage but the increment was not statistically significant (*p* < 0.05). Similar pH changes (lower than 0.22 pH units) have been previously reported as the effect of storage [8]. 

### 3.6. Lipid Oxidation

As previously shown, the reformulated patties have a high content of PUFAs and it is well known that fatty acids with a high degree of unsaturation have a high susceptibility to oxidation when incorporated into functional foods [55]. Oxidation of PUFAs is a major problem in the storage of lipid-based products, as it is responsible for the formation of off flavors, degradation processes and production of toxic compounds [56,57]. 

**Figure 1:**
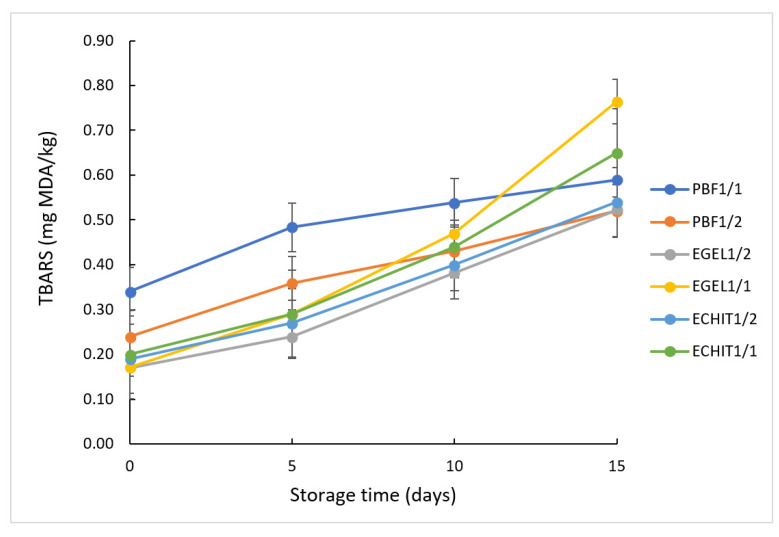
TBARS values (mg MDA/kg) of control and reformulated pork patties at 0, 5, 10 and 15 days of storage at 4 °C; PBF1/1—normal fat patties; PBF1/2—patties made by half replacement of pork backfat with water; EGEL1/2—patties made by half replacement of pork backfat with EGEL; ECHIT1/2—patties made by half replacement of pork backfat with ECHIT; EGEL1/1—patties made by full replacement of pork backfat with EGEL; ECHIT1/1—patties made by full replacement of pork backfat with ECHIT.

In order to monitor the lipid oxidation in patties, TBARS values were determined just after manufacturing (day 0) and after 5, 10 and 15 days of storage and the results are shown in Figure 1. After processing, significantly lower (*p* < 0.05) TBARS values were noticed in reformulated products as compared with the controls, probably as a result of the reduced fat content in these products in conjunction with the lower oxidative status of the incorporated oils, probably lower than that of the pork backfat. Similar findings have been previously reported by Alejandre et al. [32] in low-fat beef patties reformulated with a gelled emulsion containing algae oil or by Poyato et al. [14] in reformulated patties made by replacing pork backfat with a linseed oil gelled emulsion. They attributed this behavior to the ability of the gelled emulsion to protect the lipid fraction in the meat system based on the results of the studies comparing the stability provided by the emulsion trapping the oil, as compared with the direct incorporation of the oil in the meat product [58]. In addition, both gelatin and chitosan are gelling agents acting as a barrier to oxygen and, thus, reducing lipid oxidation [36]. Moreover, the redox-regulatory activity of chitosan is well known, which inhibits ROS production and prevents lipid oxidation [59].

The antioxidants occurring as natural constituents in the oils incorporated in the emulsion gels could also contribute to the reduction in lipid oxidation in the reformulated patties. Extra virgin olive oil represents an ideal ingredient to stabilize the *n*-3 PUFAs from algae and chia oils and to prolong their shelf life due to the large variety of antioxidant substances that it contains, including tocopherols (vitamin E), β-carotene and phenolic compounds (phenol alcohols and acids, secoiridoids, lignans and flavones) [60,61,62].

TBARS values ranged from 0.17 mg MDA/kg to 0.76 mg MDA/kg, which indicated a low lipid oxidation status in all formulations, even after 15 days of storage at 4 °C. These values are similar to those previously reported in burgers reformulated by partial or total replacement of the fat content by microparticles containing chia and linseed oils [27] or by tiger nut oil emulsion [41].

The concentration of MDA increased during the storage time in all formulations, in good agreement with the results reported by other authors [7,8]. The lowest TBARS values after 15 days of storage were found in the patties made by replacement of half of the fat content with water (PBF1/2), probably as a result of the joint effect of the diminished fat content and lower unsaturation degree. After 15 days of storage, the highest amount of MDA was found in EGEL1/1 formulation, followed by ECHIT1/1. The high content of lipids with a high degree of unsaturation present in EGEL1/1 and ECHIT1/1 may explain the higher susceptibility of lipids to oxidation during storage, despite the presence of the protective natural antioxidants in these samples. Nevertheless, all formulations had TBARS values below 1.0 mg MDA/kg, considered to be the rancidity threshold at which consumers may detect oxidized and rancid aromas [63].

### 3.7. Sensory Analysis

Consumers are interested in purchasing functional meat products if their sensorial features and price do not deviate too much from those of classic products [64]. Both control and modified patties were subjected to sensory evaluation by means of a hedonic test. Mean sensory scores for the control and reformulated patties are presented in Table 7. The color score of the PBF1/2 was found to be the highest among all formulations, followed by controls. The color scores of the reformulated patties were significantly lower (*p* < 0.05) than those of the controls. This could be in good agreement with the lower L* values and higher b* values in reformulated patties as compared with the controls as presented in Table 6. 

The taste and flavor scores were highest in patties made by half replacement of pork backfat with emulsion gels (EGEL1/2 and ECHIT1/2), while the patties made by full replacement of the fat with gels received significantly lower scores as compared with the other formulations. These ratings may be due to the characteristic fishy odor and taste of the algae oil, which were strongly felt in EGEL1/1 and ECHIT1/1. The fishy taste was also felt in EGEL1/2 and ECHIT1/2 samples, but it was faded and masked by the taste of pork fat, so the resulting taste was very pleasant. Cáceres et al. [5] found also that it was not possible to establish a clear sensory effect of the pre-emulsified fish oil on the taste of mortadella sausages. In addition, they noticed that fish oil modifies odor only when the fat level is low, otherwise fat seems to mask the fishy flavor and taste.

The texture scores were highest for ECHIT1/1 and EGEL1/1, followed by ECHIT1/2 and EGEL1/2, while the lowest scores were for the controls. These may be determined by the juiciness of the patties that is in good correlation with their moisture retention, as presented in Table 5. In addition, soy protein isolate as well as the emulsion gel stabilizer (gelatin and chitosan) act as binders, thus maintaining the tenderness of reformulated patties.

For the overall acceptability, no significant differences were found between control patties (7.50) and those reformulated by half replacement of pork backfat with emulsion gels (7.33 for EGEL1/2 and 7.67 for ECHIT1/2). However, the formulations with full replacement received significantly (*p* < 0.05) lower scores (6.50 for EGEL1/1 and 6.58 for ECHIT1/1), perhaps related to the fact that these samples were scored lower for color, taste and flavor. With regard to the stabilizer used in the gelled emulsion (gelatin and chitosan), the panelists evaluated the patties with the same proportion of emulsion gel with similar scores for all attributes.

## 4. Conclusions

The emulsion gels formulated with a mixture of olive, chia and algae oils emulsified with soy protein isolate and stabilized by gelatin (EGEL) or chitosan (ECHIT) proved to be an adequate partial or total pork backfat replacer in pork patties in view of developing new functional and healthier meat formulations. Both emulsion gels presented excellent thermal and phase separation stability over time; however, ECHIT showed higher thermal stability than EGEL. A significant decrease in the fat content was achieved in reformulated patties as compared to the controls, accompanied by the increase in the PUFAs and EPA+DHA content and the decrease in the SFA and *n*-6/*n*-3 ratio. As a result, the lipidic healthiness was improved, as demonstrated by the atherogenic (AI) and thrombogenic (TI) indices. Half replacement of pork backfat with ECHIT was the best formulation, improving the nutritional value, technological parameters and oxidative stability of pork patties without negative impact on their sensory features and acceptability.

## Figures and Tables

**Table 1:** Formulation (g/100 g) of different patties *.

	Meat	Pork Backfat	Emulsion Gel	Water	Salt
PBF1/1	73.5	20.0	-	5.0	1.5
PBF1/2	73.5	10.0	-	15.0	1.5
EGEL1/2	73.5	10.0	10.0	5.0	1.5
EGEL1/1	73.5	-	20.0	5.0	1.5
ECHIT1/2	73.5	10.0	10.0	5.0	1.5
ECHIT1/1	73.5	-	20.0	5.0	1.5

* PBF1/1—normal fat patties; PBF1/2—patties made by half replacement of pork backfat with water; EGEL1/2—patties made by half replacement of pork backfat with EGEL; ECHIT1/2—patties made by half replacement of pork backfat with ECHIT; EGEL1/1—patties made by full replacement of pork backfat with EGEL; ECHIT1/1—patties made by full replacement of pork backfat with ECHIT.

**Table 2:** Color parameters (L*—lightness, a*—redness and b*—yellowness), pH and total fluid release of gelatin (EGEL) and chitosan (ECHIT) emulsion gels at 0 and 10 days of storage at 4 °C *.

Parameter	Samples	Storage Time (Days)	
		0	10
L*	EGEL	76.92 ± 2.70 ^b,A^	79.84 ± 3.05 ^a,A^
	ECHIT	70.59 ± 3.61 ^a,A^	79.43 ± 1.77 ^a,B^
a*	EGEL	0.23 ± 0.16 ^a,B^	−0.06 ± 0.20 ^a,A^
	ECHIT	1.53 ± 0.20 ^b,B^	0.17 ± 0.08 ^b,A^
b*	EGEL	18.69 ± 2.32 ^a,A^	18.66 ± 0.57 ^b,A^
	ECHIT	20.63 ± 0.95 ^b,B^	16.90 ± 0.23 ^a,A^
pH	EGEL	7.23 ± 0.05 ^a,A^	7.38 ± 0.03 ^a,B^
	ECHIT	7.61 ± 0.07 ^b,A^	8.21 ± 0.06 ^b,B^
Total fluid release (%)	EGEL	1.34 ± 0.07 ^b,A^	1.58 ± 0.07 ^b,B^
	ECHIT	1.01 ± 0.05 ^a,A^	1.37 ± 0.08 ^a,B^

* Different lowercase letters indicate significant differences ± between emulsion gels (*p* < 0.05) for the same storage period, while different uppercase letters are indicative of significant differences between sampling times for the same emulsion gel (*p* < 0.05); EGEL—emulsion gel stabilized by gelatin; ECHIT—emulsion gel stabilized by chitosan.

**Table 3:** Proximate composition and energy values of control and reformulated pork patties *.

	PBF1/1	PBF1/2	EGEL1/2	EGEL1/1	ECHIT1/2	ECHIT1/1
Moisture (%)	53.00 ± 0.87 ^a^	61.34 ± 0.68 ^c^	57.99 ± 0.88 ^b^	61.39 ± 0.56 ^c^	57.85 ± 0.72 ^b^	61.69 ± 0.64 ^c^
Protein (%)	22.62 ± 0.63 ^a,b^	22.43 ± 0.49 ^a^	22.59 ± 0.37 ^a,b^	23.39 ± 0.48 ^b^	22.31 ± 0.65 ^a^	21.90 ± 0.54 ^a^
Fat (%)	21.47 ± 0.56 ^d^	13.72 ± 0.36 ^b^	16.91 ± 0.37 ^c^	12.91 ± 0.48 ^a^	16.53 ± 0.45 ^c^	13.57 ± 0.39 ^a,b^
Ash (%)	2.33 ± 0.10 ^a,b^	2.27 ± 0.13 ^a,b^	2.21 ± 0.11 ^a^	2.29 ± 0.11 ^a,b^	2.38 ± 0.09 ^a,b^	2.43 ± 0.14 ^b^
Energy value (kcal/100 g)	290.50 ± 5.98 ^c^	217.80 ± 4.08 ^a^	247.73 ± 5.01 ^b^	213.46 ± 4.27 ^a^	245.71 ± 4.83 ^b^	214.96 ± 4.02 ^a^
Energy from fat (kcal/100 g)	195.38 ± 5.10 ^d^	124.85 ± 3.28 ^b^	153.88 ± 3.37 ^c^	117.48 ± 4.37 ^a^	150.42 ± 4.10 ^c^	123.49 ± 3.55 ^a,b^
Fat reduction (%)	-	36.10 ± 0.45 ^c^	21.23 ± 0.33 ^a^	39.88 ± 0.67 ^d^	23.01 ± 0.69 ^b^	36.80 ± 0.57 ^c^
Energy value reduction (%)	-	25.02 ± 0.34 ^c^	14.72 ± 0.24 ^a^	26.52 ± 0.28 ^e^	15.42 ± 0.18 ^b^	26.00 ± 0.14 ^d^

* Different lowercase letters indicate significant differences between formulations (*p* < 0.05); PBF1/1—normal fat patties; PBF1/2—patties made by half replacement of pork backfat with water; ±EGEL1/2—patties made by half replacement of pork backfat with EGEL; ECHIT1/2—patties made by half replacement of pork backfat with ECHIT; EGEL1/1—patties made by full replacement of pork backfat with EGEL; ECHIT1/1—patties made by full replacement of pork backfat with ECHIT.

**Table 4:** Fatty acid profile (expressed as g/100 g of total fatty acids) and nutritional indices of control and reformulated pork patties *.

Fatty acids	PBF1/1	PBF1/2	EGEL1/2	EGEL1/1	ECHIT1/2	ECHIT1/1
Butyric (C4:0)	1.08 ± 0.05 ^a^	0.75 ± 0.03 ^b^	nd	nd	nd	nd
Caproic (C6:0)	1.67 ± 0.09 ^c^	1.53 ± 0.07 ^b^	0.07 ± 0.01 ^a^	0.04 ± 0.01 ^a^	0.08 ± 0.01 ^a^	0.05 ± 0.01 ^a^
Caprylic (C8:0)	0.68 ± 0.04 ^c^	0.68 ± 0.03 ^c^	0.12 ± 0.01 ^b^	0.04 ± 0.01 ^a^	0.12 ± 0.01 ^b^	0.05 ± 0.01 ^a^
Capric (C10:0)	0.34 ± 0.02 ^d^	0.25 ± 0.02 ^c^	0.08 ± 0.01 ^b^	0.03 ± 0.01 ^a^	0.07 ± 0.01 ^b^	0.03 ± 0.01 ^a^
Lauric (C12:0)	0.22 ± 0.02 ^a^	0.20 ± 0.01 ^a^	nd	nd	nd	nd
Myristic (C14:0)	1.70 ± 0.07 ^c^	1.63 ± 0.08 ^c^	1.00 ± 0.05 ^b^	0.45 ± 0.03 ^a^	0.97 ± 0.05 ^b^	0.47 ± 0.02 ^a^
Myristoleic (C14:1)	0.22 ± 0.02 ^a^	0.19 ± 0.02 ^a^	nd	nd	nd	nd
Pentadecanoic (C15:0)	0.85 ± 0.05 ^c^	0.61 ± 0.04 ^b^	0.08 ± 0.01 ^a^	0.05 ± 0.01 ^a^	0.08 ± 0.01 ^a^	0.08 ± 0.01 ^a^
Pentadecenoic (C15:1*n*-13)	0.92 ± 0.06 ^c^	0.78 ± 0.05 ^b^	0.18 ± 0.02 ^a^	0.21 ± 0.02 ^a^	0.17 ± 0.01 ^a^	0.16 ± 0.02 ^a^
Palmitic (C16:0)	26.51 ± 1.16 ^c^	26.17 ± 0.88 ^c^	16.17 ± 0.56 ^b^	10.35 ± 0.76 ^a^	15.72 ± 0.78 ^b^	10.72 ± 0.63 ^a^
Palmitoleic (C16:1*n*-7)	2.93 ± 0.15 ^c^	2.99 ± 0.18 ^c^	2.28 ± 0.12 ^b^	1.28 ± 0.08 ^a^	2.13 ± 0.14 ^b^	1.23 ± 0.08 ^a^
Heptadecanoic (C17:0)	0.51 ± 0.04 ^d^	0.60 ± 0.04 ^e^	0.22 ± 0.02 ^b,c^	0.20 ± 0.01 ^a,b^	0.25 ± 0.02 ^c^	0.16 ± 0.02 ^a^
Heptadecenoic (C17:1*n*-7)	0.38 ± 0.04 ^c^	0.53 ± 0.04 ^d^	0.21 ± 0.02 ^a^	0.31 ± 0. 02 ^b^	0.22 ± 0.01 ^a^	0.19 ± 0.02 ^a^
Stearic (C18:0)	11.32 ± 0.34 ^c^	11.88 ± 0.46 ^d^	6.38 ± 0.28 ^b^	3.32 ± 0.19 ^a^	6.43 ± 0.25 ^b^	3.47 ± 0.16 ^a^
Oleic (C18:1*n*-9)	37.88 ± 1.11 ^c^	40.00 ± 1.66 ^c^	33.04 ± 0.89 ^b^	26.85 ± 1.23 ^a^	31.16 ± 1.3 ^b^	27.18 ± 1.26 ^a^
Linoleic (C18:2*n*-6)	7.63 ± 0.36 ^b^	5.79 ± 0.22 ^a^	16.21 ± 0.72 ^d^	10.76 ± 0.48 ^c^	17.20 ± 0.54 ^d^	10.52 ± 0.39 ^c^
γ Linolenic (C18:3*n*-6)	0.08 ± 0.01 ^b^	0.10 ± 0.01 ^b,c^	0.05 ± 0.01 ^a^	0.04 ± 0.01 ^a^	0.12 ± 0.02 ^c^	0.22 ± 0.02 ^d^
α Linolenic (C18:3*n*-3)	0.26 ± 0.02 ^a^	0.62 ± 0.04 ^a^	10.01 ± 0.46 ^b^	21.98 ± 0.88 ^c^	10.42 ± 0.33 ^b^	22.23 ± 0.97 ^c^
Conjugated linoleic acid (CLA) (C18:3*n*-3)	0.29 ± 0.03 ^b^	0.22 ± 0.02 ^a^	0.65 ± 0.05 ^d^	0.37 ± 0.04 ^c^	0.17 ± 0.01 ^a^	0.37 ± 0.02 ^c^
Octadecatetraenoic (C18:4*n*-3)	0.60 ± 0.04 ^b^	1.10 ± 0.05 ^d^	0.53 ± 0.07 ^b^	0.24 ± 0.01 ^a^	0.85 ± 0.06 ^c^	0.22 ± 0.02 ^a^
Eicosadienoic (C20:2*n*-6)	0.74 ± 0.04 ^c^	0.73 ± 0.05 ^c^	0.45 ± 0.03 ^b^	0.24 ± 0.02 ^a^	0.46 ± 0.02 ^b^	0.20 ± 0.01 ^a^
Dihomo-γ-linolenic (C20:3*n*-6)	0.31 ± 0.02 ^a^	0.36 ± 0.02 ^b^	nd	nd	nd	nd
Erucic (C22:1*n*-9)	nd	nd	nd	nd	nd	nd
Eicosatrienoic (Mead) (C20:3*n*-3)	0.82 ± 0.05 ^b^	0.27 ± 0.03 ^a^	nd	nd	nd	nd
Arachidonic (C20:4*n*-6)	0.31 ± 0.02 ^d^	0.22 ± 0.01 ^c^	0.47 ± 0.03 ^e^	0.15 ± 0.01 ^b^	0.46 ± 0.02 ^e^	0.09 ± 0.01 ^a^
Tricosanoic (C23:0)	nd	nd	0.48 ± 0.03 ^a,b^	0.52 ± 0.04 ^b^	0.53 ± 0.04 ^b^	0.43 ± 0.02 ^a^
Docosadienoic (C22:2*n*-6)	0.10 ± 0.01 ^a^	0.18 ± 0.02 ^a^	4.46 ± 0.26 ^b^	9.57 ± 0.39 ^c^	4.88 ± 0.22 ^b^	9.37 ± 0.42 ^c^
Docosatrienoic (C22:3*n*-6)	0.09 ± 0.01 ^a^	0.14 ± 0.02 ^a^	0.65 ± 0.04 ^b^	0.79 ± 0.05 ^c^	0.70 ± 0.06 ^b^	0.84 ± 0.05 ^c^
Eicosapentaenoic (C22:5*n*-3)	0.19 ± 0.02 ^b^	0.13 ± 0.01 ^a^	0.19 ± 0.02 ^b^	0.44 ± 0.03 ^d^	0.30 ± 0.02 ^c^	0.44 ± 0.03 ^d^
Lignoceric (C24:0)	0.16 ± 0.02 ^a^	0.18 ± 0.01 ^a^	0.59 ± 0.04 ^b^	1.02 ± 0.07 ^d^	0.60 ± 0.04 ^b^	0.93 ± 0.06 ^c^
Nervonic (C24:1*n*-9)	nd	nd	0.22 ± 0.03 ^c^	0.13 ± 0.01 ^a^	0.18 ± 0.02 ^b^	0.11 ± 0.01 ^a^
Docosatetraenoic (C22:4*n*-6)	0.06 ± 0.01 ^a^	0.07 ± 0.01 ^a^	0.52 ± 0.03 ^b^	1.03 ± 0.07 ^c^	0.54 ± 0.02 ^b^	1.01 ± 0.05 ^c^
Clupanodonic (C22:5*n*-3)	nd	nd	0.47 ± 0.03 ^a^	0.99 ± 0.06 ^b^	0.54 ± 0.03 ^a^	0.97 ± 0.04 ^b^
Docosahexaenoic (C22:6*n*-3)	0.18 ± 0.02 ^a^	0.13 ± 0.01 ^a^	3.20 ± 0.18 ^b^	7.74 ± 0.34 ^c^	3.57 ± 0.21 ^b^	7.38 ± 0.42 ^c^
Other fatty acids	0.86 ± 0.05 ^c^	0.80 ± 0.06 ^b,c^	0.85 ± 0.04 ^c^	0.74 ± 0.05 ^a,b^	0.81 ± 0.07 ^b,c^	0.68 ± 0.04 ^a^
Nutritional indices						
	PBF1/1	PBF1/2	EGEL1/2	EGEL1/1	ECHIT1/2	ECHIT1/1
Σ SFA	45.04 ± 1.86 ^c^	44.48 ± 1.65 ^c^	25.19 ± 0.94 ^b^	16.02 ± 1.00 ^a^	24.85 ± 1.14 ^b^	16.39 ± 0.83 ^a^
Σ MUFA	42.33 ± 1.38 ^c^	44.49 ± 1.95 ^c^	35.93 ± 1.08 ^b^	28.78 ± 1.36 ^a^	33.86 ± 1.48 ^b^	28.87 ± 1.39 ^a^
Σ PUFA	11.74 ± 0.55 ^a^	10.16 ± 0.45 ^a^	37.91 ± 1.88 ^b^	54.38 ± 2.26 ^b^	40.33 ± 1.54 ^c^	54.08 ± 2.37 ^c^
Σ PUFA *n*-6	9.61 ± 0.49 ^b^	7.81 ± 0.36 ^a^	23.46 ± 1.11 ^c,d^	22.95 ± 0.93 ^c^	24.53 ± 0.87 ^d^	22.62 ± 0.87 ^c^
Σ PUFA *n*-3	2.13 ± 0.06 ^a^	2.35 ± 0.09 ^a^	14.45 ± 0.77 ^b^	31.43 ± 1.33 ^c^	15.80 ± 0.67 ^b^	31.46 ± 1.51 ^c^
EPA + DHA	0.37 ± 0.04 ^b^	0.26 ± 0.02 ^a^	3.39 ± 0.20 ^c^	8.18 ± 0.36 ^d^	3.87 ± 0.23 ^c^	7.82 ± 0.45 ^d^
*n*-6/*n*-3	4.51 ± 0.15 ^d^	3.32 ± 0.09 ^c^	1.62 ± 0.08 ^b^	0.73 ± 0.02 ^a^	1.55 ± 0.06 ^b^	0.72 ± 0.03 ^a^
AI	0.62 ± 0.02 ^c^	0.60 ± 0.03 ^c^	0.27 ± 0.01 ^b^	0.15 ± 0.01 ^a^	0.26 ± 0.01 ^b^	0.15 ± 0.01 ^a^
TI	1.21 ± 0.04 ^c^	1.18 ± 0.05 ^c^	0.32 ± 0.02 ^b^	0.12 ± 0.01 ^a^	0.30 ± 0.02 ^b^	0.12 ± 0.01 ^a^
h/H	1.64 ± 0.04 ^a^	1.68 ± 0.05 ^a^	3.72 ± 0.09 ^b^	6.43 ± 0.16 ^c^	3.83 ± 0.08 ^b^	6.20 ± 0.12 ^c^

* Different lowercase letters indicate significant differences between formulations (*p* < 0.05); PBF1/1—normal fat patties; PBF1/2—patties made by half replacement of pork backfat with water; EGEL1/2—patties made by half replacement of pork backfat with EGEL; ECHIT1/2—patties made by half replacement of pork backfat with ECHIT; EGEL1/1—patties made by full replacement of pork backfat with EGEL; ECHIT1/1—patties made by full replacement of pork backfat with ECHIT.

**Table 5:** Technological properties of control and reformulated pork patties *.

	PBF1/1	PBF1/2	EGEL1/2	EGEL1/1	ECHIT1/2	ECHIT1/1
Cooking loss (%)	26.6 ± 1.31 ^b^	33.7 ± 1.06 ^d^	28.82 ± 1.26 ^c^	29.32 ± 0.92 ^c^	26.62 ± 0.87 ^b^	23.14 ± 0.82 ^a^
Shrinkage (%)	4.85 ± 0.15 ^b^	4.71 ± 0.10 ^a^	4.82 ± 0.13 ^a,b^	4.84 ± 0.11 ^b^	4.87 ± 0.14 ^b^	4.87 ± 0.13 ^b^
Moisture retention (%)	38.90 ± 1.19 ^a^	40.67 ± 0.73 ^b^	41.28 ± 1.36 ^b^	43.39 ± 0.81 ^c^	42.45 ± 0.74 ^b,c^	47.41 ± 0.96 ^d^
Fat retention (%)	93.25 ± 1.46 ^c^	91.70 ± 1.64 ^c^	83.30 ± 1.37 ^b^	76.26 ± 1.69 ^a^	84.35 ± 2.01 ^b^	87.35 ± 2.15 ^b^

* Different lowercase letters indicate significant differences between formulations (*p* < 0.05); PBF1/1—normal fat patties; PBF1/2—patties made by half replacement of pork backfat with water; EGEL1/2—patties made by half replacement of pork backfat with EGEL; ECHIT1/2—patties made by half replacement of pork backfat with ECHIT; EGEL1/1—patties made by full replacement of pork backfat with EGEL; ECHIT1/1—patties made by full replacement of pork backfat with ECHIT.

**Table 6:** Color parameters (L*—lightness, a*—redness and b*—yellowness) and pH of control and reformulated pork patties at 0, 5, 10 and 15 days of storage at 4 °C *.

Parameter	Samples	Storage Time (Days)			
		0	5	10	15
L*	PBF1/1	74.63 ± 1.24 ^c,A^	74.43 ± 1.29 ^b,A^	74.39 ± 2.07 ^b,A,B^	75.31 ± 1.88 ^b,B^
	PBF1/2	76.59 ± 1.70 ^c,A^	77.10 ± 1.02 ^c,A,B^	77.48 ± 1.97 ^c,A,B^	78.75 ± 0.83 ^c,B^
	EGEL1/2	73.31 ± 3.21 ^b,A^	74.35 ± 1.24 ^b,A,B^	74.33 ± 1.11 ^b,A,B^	75.92 ± 0.40 ^b,B^
	EGEL1/1	73.39 ± 0.71 ^b,A^	73.93 ± 0.67 ^b,A,B^	74.72 ± 0.40 ^b,B^	75.60 ± 0.81 ^b,C^
	ECHIT1/2	73.23 ± 2.45 ^b,A^	73.84 ± 1.21 ^b,A,B^	74.65 ± 0.78 ^b,A,B^	75.47 ± 1.59 ^b,B^
	ECHIT1/1	68.26 ± 1.71 ^a,A^	69.03 ± 0.60 ^a,A^	69.29 ± 1.33 ^a,A^	69.90 ± 2.64 ^a,A^
a*	PBF1/1	5.79 ± 0.10 ^c,C^	4.53 ± 0.15 ^c,B^	4.47 ± 0.42 ^c,B^	3.61 ± 0.28 ^c,A^
	PBF1/2	4.88 ± 0.29 ^a,C^	3.87 ± 0.22 ^a,B^	3.61 ± 0.36 ^b,B^	3.18 ± 0.21 ^b,A^
	EGEL1/2	5.34 ± 0.47 ^b,C^	3.80 ± 0.28 ^b,B^	2.97 ± 0.18 ^a,A^	2.74 ± 0.20 ^a,A^
	EGEL1/1	5.38 ± 0.43 ^b,c,D^	4.69 ± 0.16 ^c,C^	3.85 ± 0.05 ^b,B^	3.45 ± 0.15 ^b,c,A^
	ECHIT1/2	5.38 ± 0.44 ^b,c,C^	5.13 ± 0.19 ^d,C^	4.56 ± 0.28 ^c,B^	3.64 ± 0.41 ^c,A^
	ECHIT1/1	5.55 ± 0.26 ^b,c,A^	5.65 ± 0.24 ^e,A^	5.63 ± 0.10 ^d,A^	5.62 ± 0.13 ^d,A^
b*	PBF1/1	11.15 ± 2.81 ^a,A^	11.37 ± 0.44 ^a,A^	12.07 ± 1.30 ^b,A^	12.40 ± 1.06 ^a,b,A^
	PBF1/2	11.15 ± 0.60 ^a,A^	11.83 ± 0.35 ^a,b,B^	10.99 ± 0.28 ^a,A^	12.14 ± 0.17 ^a,B^
	EGEL1/2	11.90 ± 0.77 ^a,b,A^	12.27 ± 0.47 ^b,A,B^	12.74 ± 0.11 ^b,B,C^	13.10 ± 0.64 ^b,c,C^
	EGEL1/1	13.13 ± 0.74 ^b,A,B^	13.60 ± 0.49 ^d,B^	12.85 ± 0.25 ^b,A^	13.55 ± 0.07 ^c,d,B^
	ECHIT1/2	12.23 ± 0.99 ^a,b,A^	12.20 ± 0.43 ^b,A^	12.95 ± 0.65 ^b,A,B^	13.25 ± 0.79 ^c,B^
	ECHIT1/1	12.59 ± 0.43 ^a,b,A^	13.06 ± 0.30 ^c,A,B^	13.44 ± 0.34 ^b,B^	14.28 ± 0.93 ^d,C^
pH	PBF1/1	5.89 ±0.13 ^a,A^	5.98 ± 0.08 ^a,b,A^	6.00 ± 0.11 ^a,b,A^	6.07 ± 0.16 ^a,b,A^
	PBF1/2	5.79 ± 0.09 ^a,A^	5.85 ± 0.12 ^a,A^	5.87 ± 0.14 ^a,A^	5.89 ± 0.11 ^a,A^
	EGEL1/2	5.90 ± 0.12 ^a,A^	5.92 ± 0.14 ^a,b,A^	5.94 ± 0.16 ^a,b,A^	5.96 ± 0.14 ^a,A^
	EGEL1/1	5.94 ± 0.09 ^a,A^	5.96 ± 0.15 ^a,b,A^	5.98 ± 0.11 ^a,b,A^	6.01 ± 0.14 ^a,b,A^
	ECHIT1/2	5.98 ± 0.12 ^a,A^	6.09 ± 0.10 ^b,c,A^	6.15 ± 0.13 ^b,c,A^	6.20 ± 0.14 ^b,c,A^
	ECHIT1/1	6.28 ± 0.13 ^b,A^	6.32 ± 0.18 ^c,A^	6.36 ± 0.10 ^c,A^	6.39 ± 0.09 ^c,A^

* Different lowercase letters indicate significant differences between patty samples (*p* < 0.05) for the same storage period, while different uppercase letters are indicative of significant differences between sampling times for the same patty formulation (*p* < 0.05); PBF1/1—normal fat patties; PBF1/2—patties made by half replacement of pork backfat with water; EGEL1/2—patties made by half replacement of pork backfat with EGEL; ECHIT1/2—patties made by half replacement of pork backfat with ECHIT; EGEL1/1—patties made by full replacement of pork backfat with EGEL; ECHIT1/1—patties made by full replacement of pork backfat with ECHIT.

**Table 7:** Sensory features and overall acceptability scores of control and reformulated pork patties *.

	PBF1/1	PBF1/2	EGEL1/2	EGEL1/1	ECHIT1/2	ECHIT1/1
Color	7.25 ± 0.62 ^b^	7.92 ± 0.67 ^c^	6.67 ± 0.49 ^a^	6.33 ± 0.65 ^a^	6.83 ± 0.72 ^a,b^	6.42 ± 0.51 ^a^
Taste	6.83 ± 0.72 ^a,b^	7.17 ± 0.72 ^b,c^	7.92 ± 0.51 ^d^	6.67 ± 0.89 ^a,b^	7.67 ± 0.65 ^c,d^	6.50 ± 0.52 ^a^
Flavor	7.33 ± 0.65 ^b^	7.42 ± 0.67 ^b^	7.58± 0.51 ^b^	6.58 ± 0.67 ^a^	7.67 ± 0.49 ^b^	6.50 ± 0.52 ^a^
Texture	6.75 ± 0.62 ^a^	7.00 ± 0.74 ^a,b^	7.42 ± 0.67 ^b,c^	7.83 ± 0.83 ^c,d^	7.58 ± 0.51 ^c^	8.17 ± 0.72 ^d^
Overall acceptability	7.50 ± 0.52 ^b^	7.75 ± 0.45 ^b^	7.33 ± 0.49 ^b^	6.50 ± 0.67 ^a^	7.67 ± 0.49 ^b^	6.58 ± 0.51 ^a^

* Different lowercase letters indicate significant differences between formulations (*p* < 0.05); PBF1/1—normal fat patties; PBF1/2—patties made by half replacement of pork backfat with water; EGEL1/2—patties made by half replacement of pork backfat with EGEL; ECHIT1/2—patties made by half replacement of pork backfat with ECHIT; EGEL1/1—patties made by full replacement of pork backfat with EGEL; ECHIT1/1—patties made by full replacement of pork backfat with ECHIT.

## Data Availability

Data is contained within the article or Supplementary Material.

## Supplementary Materials

The following supporting information can be downloaded at: https://www.mdpi.com/article/10.3390/foods12030519/s1, Figure S1. Appearance of gelatin (EGEL) and chitosan (ECHIT) emulsion gels; Figure S2. Appearance of control and reformulated patties immediately after processing.
