# Supplementary material for: Effects of Pork Backfat Replacement with Emulsion Gels Formulated with a Mixture of Olive, Chia and Algae Oils on the Quality Attributes of Pork Patties
Source: Foods. 2023 Jan 23;12(3):519. doi: 10.3390/foods12030519 (PMC9914842; doi:10.3390/foods12030519)
Supplement: Supplementary file 1 [file foods-12-00519-s001.zip › foods-2173858-supplementary.pdf]

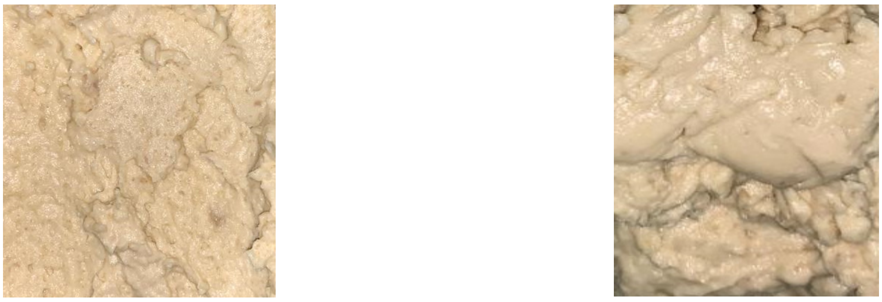

Figure S1. Appearance of gelatin (EGEL) and chitosan (ECHIT) emulsion gels; EGEL – emulsion gel stabilized by gelatin; ECHIT – emulsion gel stabilized by chitosan.

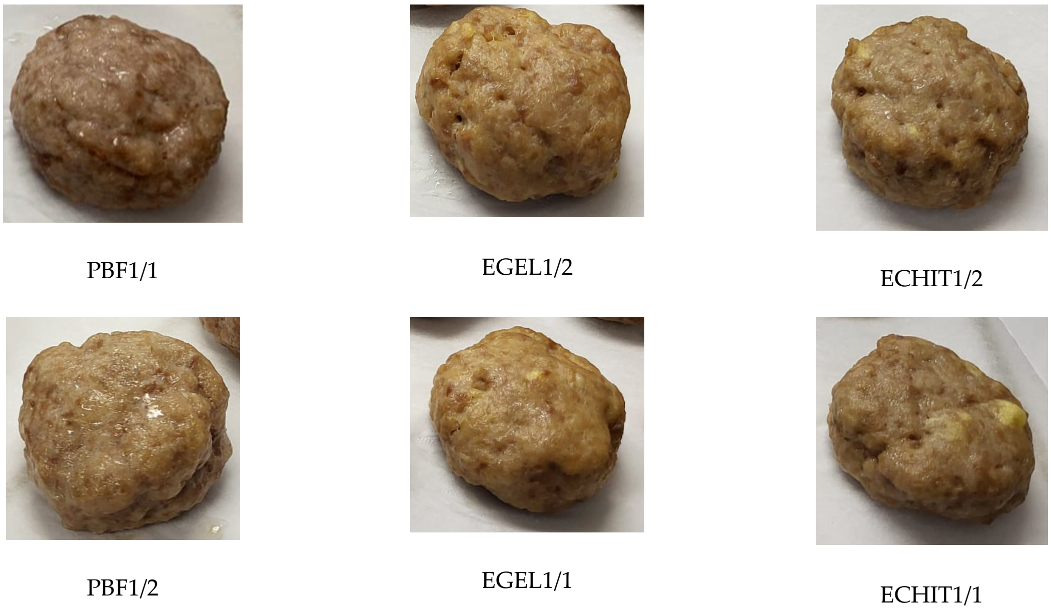

Figure S2. Appearance of control and reformulated patties immediately after processing; PBF1/1 – normal fat patties; PBF1/2 - patties made by half replacement of pork backfat with water; EGEL1/2 – patties made by half replacement of pork backfat with EGEL; ECHIT1/2 – patties made by half replacement of pork backfat with ECHIT; EGEL1/1 – patties made by full replacement of pork backfat with EGEL; ECHIT1/1 – patties made by full replacement of pork backfat with ECHIT.
